# Dietary *Enteromorpha* Polysaccharides Supplementation Improves Breast Muscle Yield and Is Associated With Modification of mRNA Transcriptome in Broiler Chickens

**DOI:** 10.3389/fvets.2021.663988

**Published:** 2021-04-16

**Authors:** Yue Zhao, Balamuralikrishnan Balasubramanian, Yan Guo, Sheng-Jian Qiu, Rajesh Jha, Wen-Chao Liu

**Affiliations:** ^1^Department of Animal Science, College of Coastal Agricultural Sciences, Guangdong Ocean University, Zhanjiang, China; ^2^Department of Food Science and Biotechnology, College of Life Science, Sejong University, Seoul, South Korea; ^3^Department of Human Nutrition, Food and Animal Sciences, College of Tropical Agriculture and Human Resources, University of Hawaii at Manoa, Honolulu, HI, United States

**Keywords:** broilers, breast muscle, *Enteromorpha* polysaccharides, RNA-Seq, transcriptom

## Abstract

The present study evaluated the effects of dietary supplementation of *Enteromorpha* polysaccharides (EP) on carcass traits of broilers and potential molecular mechanisms associated with it. This study used RNA-Sequencing (RNA-Seq) to detect modification in mRNA transcriptome and the cognate biological pathways affecting the carcass traits. A total of 396 one-day-old male broilers (Arbor Acres) were randomly assigned to one of six dietary treatments containing EP at 0 (CON), 1000 (EP_1000), 2500 (EP_2500), 4000 (EP_4000), 5500 (EP_5500), and 7000 (EP_7000) mg/kg levels for a 35-d feeding trial with 6 replicates/treatment. At the end of the feeding trial, six birds (one bird from each replicate cage) were randomly selected from each treatment and slaughtered for carcass traits analysis. The results showed that the dietary supplementation of EP_7000 improved the breast muscle yield (*p* < 0.05). Subsequently, six breast muscle samples from CON and EP_7000 groups (three samples from each group) were randomly selected for RNA-Seq analysis. Based on the RNA-Seq results, a total of 154 differentially expressed genes (DEGs) were identified (*p* < 0.05). Among the DEGs, 112 genes were significantly upregulated, whereas 42 genes were significantly down-regulated by EP_7000 supplementation. Gene Ontology enrichment analysis showed that the DEGs were mainly enriched in immune-related signaling pathways, macromolecule biosynthetic, DNA-templated, RNA biosynthetic, and metabolic process (*p* < 0.05). Kyoto Encyclopedia of Genes and Genomes pathway analysis showed that the DEGs were enriched in signaling pathways related to viral infectious diseases and cell adhesion molecules (*p* < 0.05). In conclusion, dietary inclusion of EP_7000 improves the breast muscle yield, which may be involved in improving the immunity and the cell differentiation of broilers, thus promoting the muscle growth of broilers. These findings could help understand the molecular mechanisms that enhance breast muscle yield by dietary supplementation of EP in broilers.

## Introduction

The antibiotics growth-promoters (AGP) have been widely used in poultry production to maintain high productivity and improve economic efficiency. However, abuse of AGP might bring antibiotic resistance in poultry and antibiotic residues in poultry products, leading to food safety and public health concerns (1, 2). Therefore, the search for in-feed antibiotic substitutes has become a research focus in recent years. Natural bioactive compounds possess several beneficial biological activities and maybe potential substitutes for antibiotics (3–5). Natural polysaccharides exert many functional benefits and improve broiler's growth performance and gut health (6–8). Thus, the natural polysaccharides can be used as growth promoters in broilers. The marine environment has rich sources of natural polysaccharides, and marine-derived polysaccharides have been confirmed to exert various biological functions, such as anti-oxidant, and anti-inflammatory responses (9). For instance, chitosan and chito-oligosaccharides improved the carcass traits, immunity, and anti-oxidant effects in broilers (10–13) and promoted beneficial gut microbiota while modulating metabolic pathways (8). However, to the best of our knowledge, there is no report evaluating the effects of dietary seaweed polysaccharides on carcass traits and ts he mRNA transcriptome of broilers.

Carcass traits are indicators of differences in the amount of nutrients deposited in tissues (14). Slaughter and whole net carcass yield can measure the performance of meat yield (15, 16). Meanwhile, the breast and leg muscle percentage can determine the meat yield of broilers (17). Therefore, the improvement of breast muscle yield is considered to be significant in the poultry industry. Knowing the mechanism associated with it will further help to develop a nutrition program. RNA-Sequencing (RNA-Seq) analysis has emerged as a powerful technology for transcriptome analysis to detect the whole transcriptional level of species (18). New transcripts could be found in the structure and expression level of the enriched single-chain mRNA by RNA-Seq. Also, the expression level of the low abundance gene was verified using RNA-Seq with desired results (19). At present, the RNA-Seq technique is of great significance to explore gene expression and regulation mechanisms at the transcriptional level and is widely used in life sciences research (20).

*Enteromorpha* is one of the largest green seaweeds, widely distributed in various sea. It contains abundant polysaccharides and has been confirmed to exert several biological functions, such as antitumor, antioxidation, and antiviral activities (21). Dietary inclusion of *Enteromorpha* polysaccharides (EP) enhanced the growth performance, antioxidation, and immunity in broilers (22). We conducted series of studies to evaluate different roles of dietary EP in various parameters of broilers. Dietary supplementation of EP improved the anti-oxidant performance in laying hens (9) and growth performance and intestinal barrier function in broilers (23). Similarly, our previous study found that the supplementation of EP mitigated AFB_1_-induced toxicological effect of broilers (24). Also, it has been reported that natural dietary polysaccharides could improve the carcass traits in broilers (25, 26). However, to date, the effects of dietary EP on carcass traits of broilers, especially the underlying molecular mechanism, are unknown. Hence, the present study was conducted to evaluate the effect of dietary supplementation with graded levels of EP on carcass traits in broilers and to explore the molecular mechanism of mRNA transcriptome by using the RNA-Seq technique.

## Materials and Methods

### Source of EP

The EP were extracted from the *Enteromorpha* by Qingdao Haida Biotechnology Co., Ltd. (Qingdao, Shandong, China), with ≥ 48% purity and a molecular weight of 4,929 Da. The EP are water-soluble sulfated polysaccharides obtained from the natural green alga *Enteromorpha* by enzymatic extraction, purification, concentration, and spray drying. Briefly, after crushing the algae, the algal powders are soaked in water. Then the water extracts of the algae are subjected to stepwise enzymatic treatment with pectinase, cellulase, and papain. Then the enzymes are inactivated, centrifugal concentrated, precipitated with ethanol, and finally spray dried to obtain the EP used in this study. Based on the analysis by high-performance liquid chromatography (HPLC), the polysaccharides were mainly consisting of rhamnose (Rha), glucuronic acid (GlcA), glucose (Glc), galactose (Gal), and xylose (Xyl) monosaccharides. The molar percentage of monosaccharides in the EP were as follows: Rha 40.6%, GlcA 9.3%, Glc 38.2%, Gal 5.6%, and Xyl 6.3%.

### Experimental Design, Birds, and Diets

A total of 396 1-day-old male Arbor Acres broiler chicks (initial body weight 44.65 ± 0.56 g) were obtained from a commercial hatchery (Nanning, Guangxi, China). The chicks were randomly allocated to 1 of 6 dietary treatments (6 replicates per treatment, with 11 broilers per cage), and the study was conducted for 35 day. Dietary treatments were as follows: basal diets supplemented with EP at 0 (CON), 1000 (EP_1000), 2500 (EP_2500), 4000 (EP_4000), 5500 (EP_5500), and 7000 (EP_7000) mg/kg of feed. The mash form basal diet was formulated (Table 1) to meet or exceed the nutrient requirements of broiler chickens (27) in two phases-starter (1–21 day) and finisher (22–35 day). The broilers were grown in a temperature-controlled room at 33 ± 1°C for the first 3 day and then gradually reduced by 3°C per week until reaching 24°C and maintaining humidity 65% for the rest of the study period. The birds had free access to feed and water all the time during the study period.

**Table 1:** Basal diet composition (as-fed basis).

**Items**	**D 1–21**	**D 22–35**
**Ingredients (%)**		
Corn	57.2	60.74
Soybean meal, CP 45%	29.24	25.03
Corn gluten meal, CP 60%	4.4	3.83
Soybean oil	3.41	5
Limestone	0.91	1.02
Dicalcium phosphate	2.07	1.93
Salt	0.32	0.37
Methionine, 99%	0.33	0.37
Lysine-HCI, 24%	1.68	1.28
Threonine, 98.5%	0.18	0.18
Vitamin premix^a^	0.06	0.05
Trace mineral premix^b^	0.1	0.1
Choline, 50%	0.1	0.1
**Calculated values**		
ME (kcal/kg)	3020	3200
CF (%)	6.3	7.5
Lys (%)	1.5	1.2
CP (%)	22.2	20.07
Met (%)	0.65	0.64
Met+Cys (%)	1.37	1.41
Ca (%)	0.9	0.95
Total P (%)	0.71	0.66

a *Provided per kilogram of diet: 15,000 IU of vitamin A, 3,750 IU of vitamin D3, 37.5 mg of vitamin E, 2.55 mg of vitamin K3, 3 mg of thiamin, 7.5 mg of riboflavin, 4.5 mg of vitamin B6, 24 μg of vitamin B12, 51 mg of niacin, 1.5 mg of folic acid, 0.2 mg of biotin, and 13.5 mg of pantothenic acid*.

b *Provided per kilogram of diet: 37.5 mg of Zn, 37.5 mg of Mn, 37.5 mg of Fe, 3.75 mg of Cu, 0.83 mg of I, and 62.5 mg of S*.

### Carcass Traits Determination

At the end of the feeding trial, all birds were fasted for 12 h, and six birds (one bird from each replicate cage) were randomly selected from each treatment. Then the birds were weighed and slaughtered. The defeathered carcass, including the head and feet, was weighed as the carcass weight. The carcass was then eviscerated manually and weighed as the semi-eviscerated weight, which was measured as the carcass weight after removed the esophagus, trachea, spleen, gastrointestinal tract, crop, gallbladder, pancreas, and gonads. Eviscerated weight was measured as the semi-eviscerated weight after removed the head, feet, liver, heart, glandular stomach, gizzard, and abdominal fat. Finally, the carcass, semi-eviscerated carcass, eviscerated carcass, breast muscle, leg muscle, and abdominal fat yield was calculated as a percentage of live body weight. The detailed determination of carcass characteristics was done as follows.

Carcass yield (%) = (carcass weight/live body weight) × 100%;
Semi-eviscerated carcass yield (%) = (semi-eviscerated weight/live body weight) × 100%;
Eviscerated carcass yield (%) = (eviscerated weight/live body weight) × 100%;
Breast muscle yield (%) = (breast muscle weight on both sides/live body weight) × 100%;
Leg muscle yield (%) = (leg muscle weight on both sides/live body weight) × 100%;
Abdominal fat yield (%) = (abdominal fat weight/live body weight) × 100%.

### Transcriptome Analysis

Based on the carcass traits data (Table 3), six breast muscle samples with significant differences between two treatments (CON and EP_7000 groups, three samples in each group) were randomly selected and sent for RNA-Seq analysis (Shanghai Majorbio Biological Co., Ltd., Shanghai, China). Total RNA was isolated from the breast muscle samples using TRIzol Reagent (Invitrogen, Carlsbad, CA, USA) according to the manufacturer's recommendations. For each of samples, 10 μg of RNA was used for RNA-seq library preparation by using the stranded Total RNA kit (Illumina TruSeq®) followed by the kit's directions. The mRNA isolated from total RNA is used for the analysis of transcription information. Then, randomly interrupt the mRNA, add fragmentation buffer to break it into small fragments of about 300 bp. A six-base-pair random hexamer was added, and the mRNA was used as a template to reversely synthesize a single-strand cDNA, which was then synthesized into a two-stranded stable structure. After purification and enrichment, all libraries were sequenced using an Illumina HiSeq 2000 (Illumina, San Diego, CA, USA). After obtaining the Read Counts of the transcript, analyze the expression difference of the transcript between samples for the multi-sample project. The differentially expressed genes (DEGs) among different groups were analyzed using EdgeR software (Majorbio, Shanghai, China), *p*-values were adjusted (padj) using the Benjamini-Hochberg approach for controlling the false discovery rate. The GOATOOLS were used to perform Gene Ontology (GO) enrichment analysis on the transcripts in the gene set. When the corrected *p* < 0.05, the GO function is considered to be significantly enriched. The R Programming Language was used to write a script to perform Kyoto Encyclopedia of Genes and Genomes (KEGG) pathway enrichment analysis on the transcripts in the gene set. When the *p* < 0.05, it is considered that the KEGG pathway is significantly enriched.

Six DEGs were selected to verify the RNA-Seq results by using quantitative real- time PCR (qPCR). The description of the specific primers used for qPCR are presented in Table 2 (Primer Express 3.0 software, Applied Biosystems, Foster City, CA, USA). Primers were developed by Sangon Biotech Co., Ltd. (Shanghai, China), and the β-actin was used as the reference gene. The qPCR reactions were performed with a CFX-96 Real-Time PCR Detection System (BioRad, Hercules, CA, USA). It was carried out in a total volume of 20 μl, including 10 μL SYBR® Premix Ex Taq II (Tli RNaseH Plus), 2 μl cDNA template, 1 μl of each primer (forward and reverse primers), and 6 μl DEPC treated water. The DEPC treated water for the replacement of cDNA template was used as a negative control. The PCR program was as follows: 95°C for 30 s, followed by 40 cycles of 95°C for 10 s, 30 s under T_m_ temperature, and 72°C for 15 s. Each sample was tested in triplicate. The relative mRNA expression levels of the six target genes were calculated using the 2^−ΔΔCt^ method.

**Table 2:** Description of primers used in qPCR verification.

**Genes**	**Primer sequence (5'-3')**	**Product size (bp)**	**Annealing temperature (°C)**	**Accession No**.
*ASB2*	F: CCTCCTACCTCTTCACATAGCA	176	55.0	XM_015287761.2
	R: TCACCTCATAGCCAGCATCAA			
*FBXO32*	F: CGTTGTAAGGCTGTTGGA	129	50.0	NM_001030956.1
	R: CTGATGTTCTGCTGGTCTT			
*ARNTL*	F: ACTACGCAGACCAACAAG	111	50.0	XM_015286335.2
	R: CCAAGGATGCCAGTTCAT			
*CCL19*	F: TCTCTGCCTTAGTCTCCTG	132	52.0	NM_001302168.1
	R: AGCTGCATCCTGTAGTCC			
*POU2AF1*	F: CCAGTGAAGGAGCTATTGAA	112	52.0	NM_204175.2
	R: CCATTGGTGAGTAGGAAGG			
*PER3*	F: TGCTACAAGAAGTCAGGAAG	128	51.0	NM_001289779.2
	R: TGCTACCAAGGCTACCATA			
*β-actin*	F: GCGTGACATCAAGGAGAAGC	187	60.0	NM_205518.1
	F: GGACTCCATACCCAAGAAAGAT			

### Statistical Analysis

The carcass data were statistically analyzed using general linear model procedures of SAS (SAS V9.2, SAS Institute Inc., Cary, NC, USA) with a pen as experimental unit. Orthogonal polynomial contrasts of the carcass data were used to test the linear, quadratic, and cubic effects of the increasing levels of dietary EP. The DEGs analysis of transcriptome was done using EdgeR software (Majorbio, Shanghai, China), genes enrichment pathway was analyzed using GOATOOLS software (GO analysis, Majorbio, Shanghai, China) and Majorbio software (KEGG analysis, Majorbio, Shanghai, China). The probability value of <0.05 was considered to be statistically significant, and 0.05 ≤ *p* < 0.10 was considered as a tendency.

## Results

### Carcass Traits

As shown in Table 3, graded dietary levels of EP supplementation had no significant effects on carcass, semi-eviscerated carcass, eviscerated carcass, leg muscle, and abdominal fat yield (*p* > 0.05). However, broilers in EP_7000 group had a significantly higher breast muscle yield than the CON group (*p* < 0.05).

**Table 3:** Effects of dietary supplementation of *Enteromorpha* polysaccharides (EP) on carcass traits of broilers on d 35.

**Dietary EP levels (mg/kg)**	**Carcass yield, %**	**Semi-eviscerated carcass yield, %**	**Eviscerated carcass yield, %**	**Breast muscle yield, %**	**Leg muscle yield, %**	**Abdominal fat yield, %**
0	91.62	78.05	62.42	**15.29**^a^	19.75	2.35
1000	91.75	78.77	63.41	16.18^ab^	20.55	2.37
2500	90.47	78.26	62.29	15.82^ab^	21.26	2.21
4000	91.71	79.96	64.44	16.55^ab^	19.98	2.65
5500	91.41	79.02	62.37	17.42^ab^	20.91	2.55
7000	91.72	80.22	63.67	**18.41^b^**	20.92	2.22
SEM^1^	0.49	0.80	0.94	0.98	0.69	0.35
Contrast	*p*-value					
Linear	0.590	0.155	0.252	0.621	0.654	0.646
Quadratic	0.188	0.543	0.547	0.905	0.144	0.562
Cubic	0.089	0.345	0.213	0.665	0.545	0.622

1 *SEM, standard error of mean*.

a−b *Values in the same line with different small letter superscripts mean significant difference (p < 0.05), while with same or no letter superscripts mean no significant difference (p > 0.05)*.

### Transcriptome Analysis

**Figure 1:**
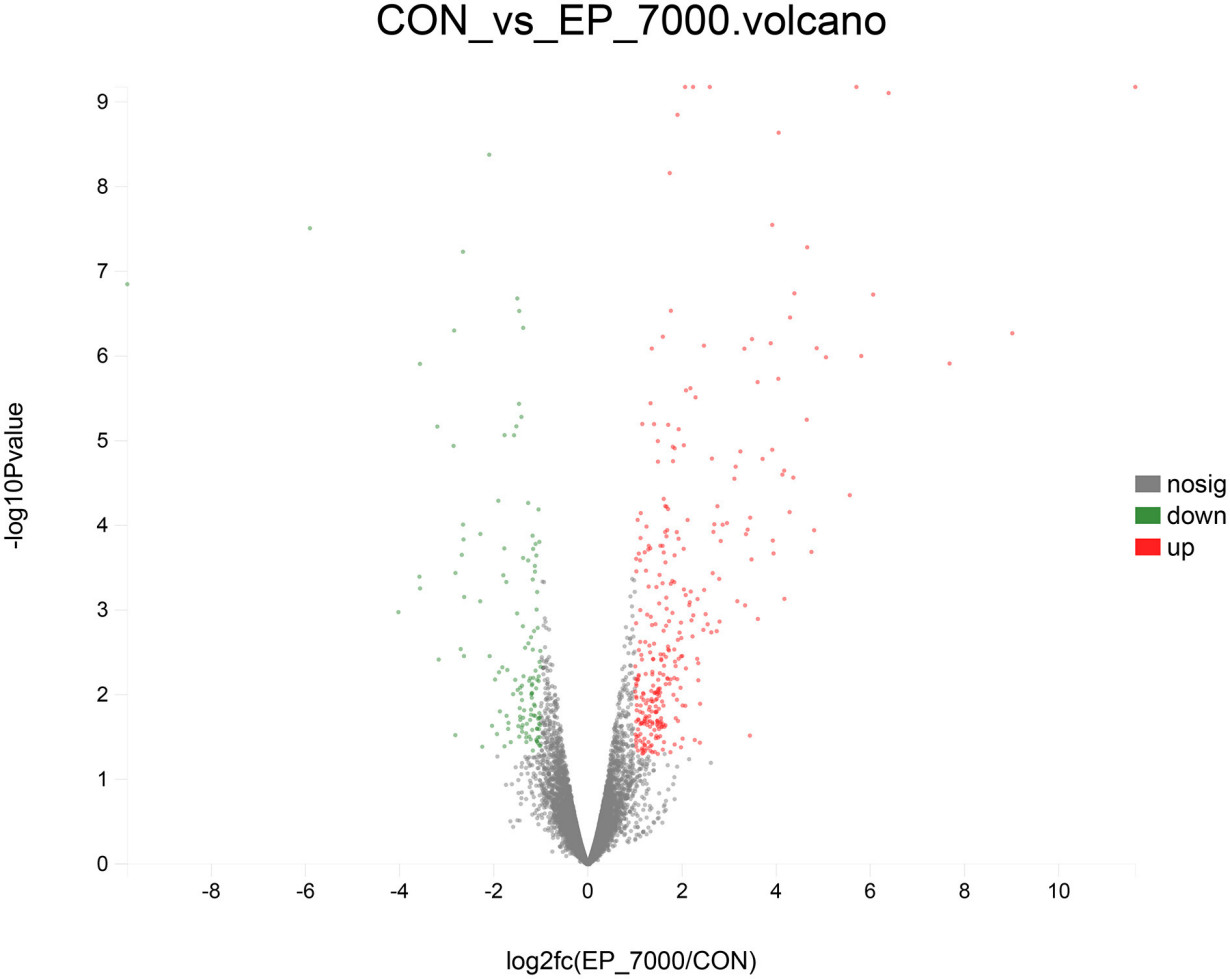
Volcano plot of differentially expressed genes of broiler fed with control and EP_7000 supplemented diets. CON, dietary supplementation of *Enteromorpha* polysaccharides (EP) at 0 mg/kg; EP_7000, dietary supplementation of *Enteromorpha* polysaccharides (EP) at 7000 mg/kg; nosig, no significant.

**Figure 2:**
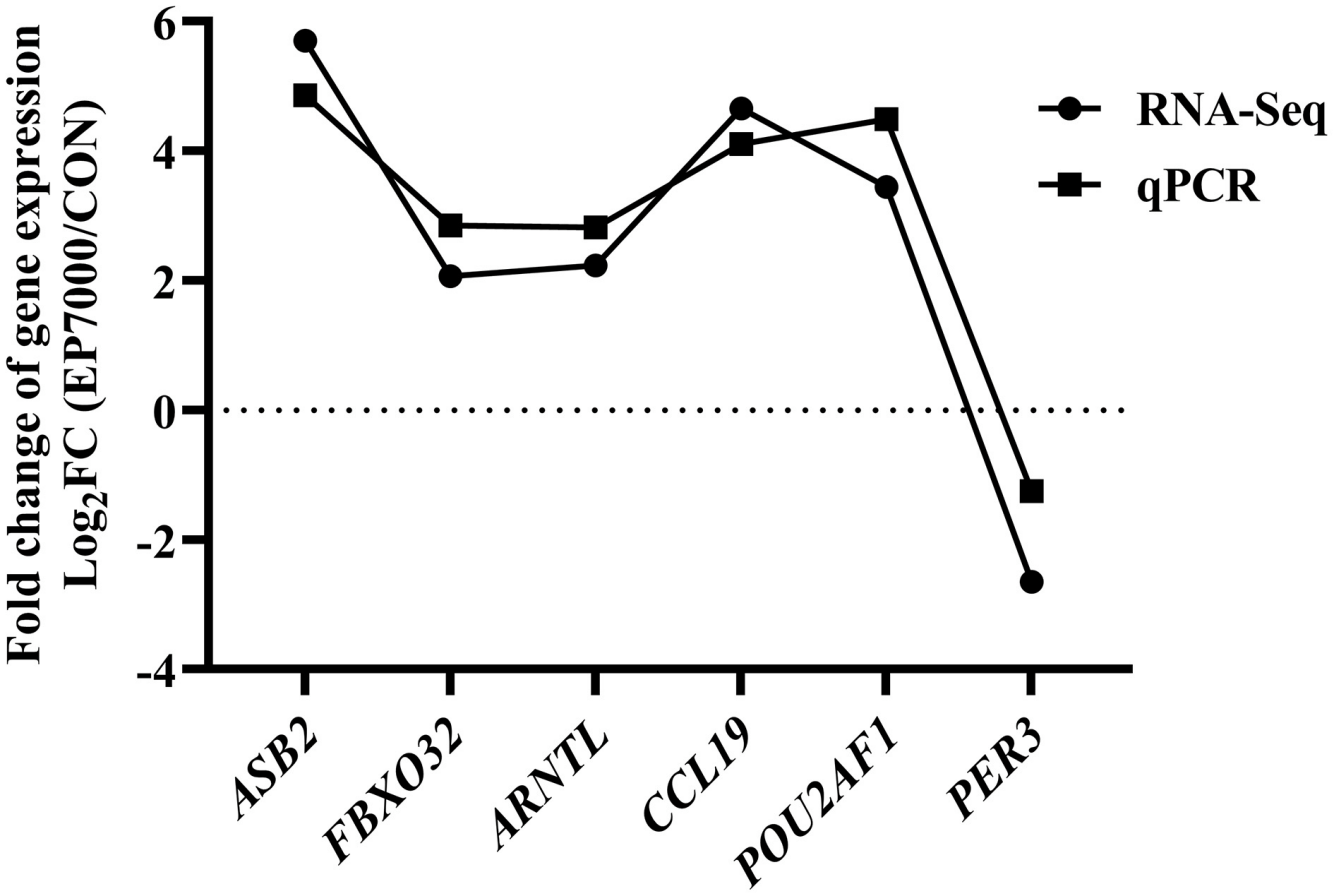
Validation of RNA-Seq results by qPCR. CON, dietary supplementation of *Enteromorpha* polysaccharides (EP) at 0 mg/kg; EP_7000, dietary supplementation of *Enteromorpha* polysaccharides (EP) at 7000 mg/kg.

The results of DEGs are presented in Figure 1 and Supplementary Table 1, and the selected DEGs (top 30 upregulated and 30 down-regulated genes) are presented in Tables 4, 5. Between the CON and EP_7000 groups, a total of 154 genes (*p*-adjust < 0.05) were differentially expressed. Among the identified 154 DEGs, 112 genes were significantly upregulated, and 42 genes were significantly down-regulated by EP_7000 supplementation. The six selected DEGs showed a consistent expression trend between the RNA-Seq and qPCR, suggesting that the results of RNA-Seq are reliable (Figure 2).

**Table 4:** The 30 most upregulated differentially expressed genes in the broiler chickens fed *Enteromorpha* polysaccharides compared to control group^a^.

**Gene ID**	**Gene name**	**Gene description**	**Fold change Log2FC (EP_7000/CON)**	***P*-value (adjusted)**
ENSGALG00000045075	-	-	11.63	0.0000
ENSGALG00000009479	-	-	9.017	0.0000
ENSGALG00000014962	FAM26F	Family with sequence similarity 26 member F	7.684	0.0000
ENSGALG00000041421	-	-	6.389	0.0000
ENSGALG00000045392	-	-	6.061	0.0000
ENSGALG00000013548	GZMA	Granzyme A	5.808	0.0000
ENSGALG00000010881	ASB2	Ankyrin repeat and SOCS box containing 2	5.705	0.0000
ENSGALG00000042285	-	-	5.058	0.0000
ENSGALG00000033171	TGM4	Transglutaminase 4	4.861	0.0000
ENSGALG00000028256	CCL19	C-C motif chemokine ligand 19	4.659	0.0000
ENSGALG00000045085	-	-	4.388	0.0000
ENSGALG00000021139	-	-	4.296	0.0000
ENSGALG00000016400	RSAD2	Radical S-adenosyl methionine domain containing 2	4.053	0.0000
ENSGALG00000011190	-	-	4.046	0.0010
ENSGALG00000013575	-	-	3.917	0.0000
ENSGALG00000013723	OASL	2'-5'-Oligoadenylate synthetase like	3.885	0.0000
ENSGALG00000032340	-	-	3.606	0.0010
ENSGALG00000011551	JCHAIN	Joining chain of multimeric IgA and IgM	3.487	0.0000
ENSGALG00000041944	-	-	3.325	0.0000
ENSGALG00000015779	PM20D2	Peptidase M20 domain containing 2	2.59	0.0000
ENSGALG00000039269	-	-	2.466	0.0000
ENSGALG00000005378	ARNTL	Aryl hydrocarbon receptor nuclear translocator like	2.235	0.0000
ENSGALG00000014297	IRF7	Interferon regulatory factor 7	2.178	0.0010
ENSGALG00000006138	-	-	2.085	0.0010
ENSGALG00000041202	FBXO32	F-box protein 32	2.066	0.0000
ENSGALG00000036356	YEATS2	YEATS domain containing 2	1.904	0.0000
ENSGALG00000016456	Lpin1	Lipin 1	1.763	0.0000
ENSGALG00000041787	PLA2G15	Phospholipase A2 group XV	1.740	0.0000
ENSGALG00000020975	TMEM233	Transmembrane protein 233	1.593	0.0000
ENSGALG00000015286	SESN1	Sestrin 1	1.358	0.0000

a *Dietary supplementation of Enteromorpha polysaccharides at 7,000 mg/kg in treatment group as compared to 0 mg/kg in control group*.

**Table 5:** The 30 most down-regulated differentially expressed genes in the broiler chickens fed *Enteromorpha* polysaccharides compared to control group^a^.

**Gene ID**	**Gene name**	**Gene description**	**Fold change Log2FC (EP_7000/CON)**	***P*-value (adjusted)**
ENSGALG00000040636	-	-	−9.784	0.000
ENSGALG00000035177	-	-	−5.904	0.000
ENSGALG00000034135	-	-	−3.567	0.000
ENSGALG00000028560	-	-	−3.198	0.002
ENSGALG00000029186	-	-	−2.853	0.002
ENSGALG00000027874	CHAC1	ChaC glutathione specific gamma-glutamylcyclotransferase 1	−2.840	0.000
ENSGALG00000032091	-	-	−2.678	0.022
ENSGALG00000000573	PER3	Period circadian clock 3	−2.653	0.000
ENSGALG00000039863	-	-	−2.652	0.013
ENSGALG00000039919	-	-	−2.642	0.017
ENSGALG00000009495	FGFR2	Fibroblast growth factor receptor 2	−2.284	0.016
ENSGALG00000011084	-	-	−2.095	0.000
ENSGALG00000032602	-	-	−1.902	0.008
ENSGALG00000005095	SLC25A25	Solute carrier family 25 member 25	−1.775	0.020
ENSGALG00000042810	-	-	−1.770	0.002
ENSGALG00000029410	-	-	−1.568	0.002
ENSGALG00000014776	-	-	−1.519	0.002
ENSGALG00000011291	NR1D2	Nuclear receptor subfamily 1 group D member 2	−1.499	0.000
ENSGALG00000001918	DNAJB5	DnaJ heat shock protein family (Hsp40) member B5	−1.463	0.001
ENSGALG00000027472	TMEM38B	Transmembrane protein 38B	−1.458	0.000
ENSGALG00000000123	-	-	−1.412	0.001
ENSGALG00000007300	-	-	−1.376	0.023
ENSGALG00000008308	BHLHE40	Basic helix-loop-helix family member e40	−1.374	0.000
ENSGALG00000000107	TRIM7	Tripartite motif containing 7	−1.268	0.009
ENSGALG00000008266	COL4A6	Collagen type IV alpha 6 chain	−1.175	0.016
ENSGALG00000015663	HSDL2	Hydroxysteroid dehydrogenase like 2	−1.163	0.020
ENSGALG00000015704	-	-	−1.110	0.019
ENSGALG00000028140	CNTFR	Ciliary neurotrophic factor receptor	−1.094	0.022
ENSGALG00000036530	-	-	−1.048	0.010
ENSGALG00000001986	-	-	−1.030	0.018

a *Dietary supplementation of Enteromorpha polysaccharides at 7,000 mg/kg in treatment group as compared to 0 mg/kg in control group*.

**Figure 3:**
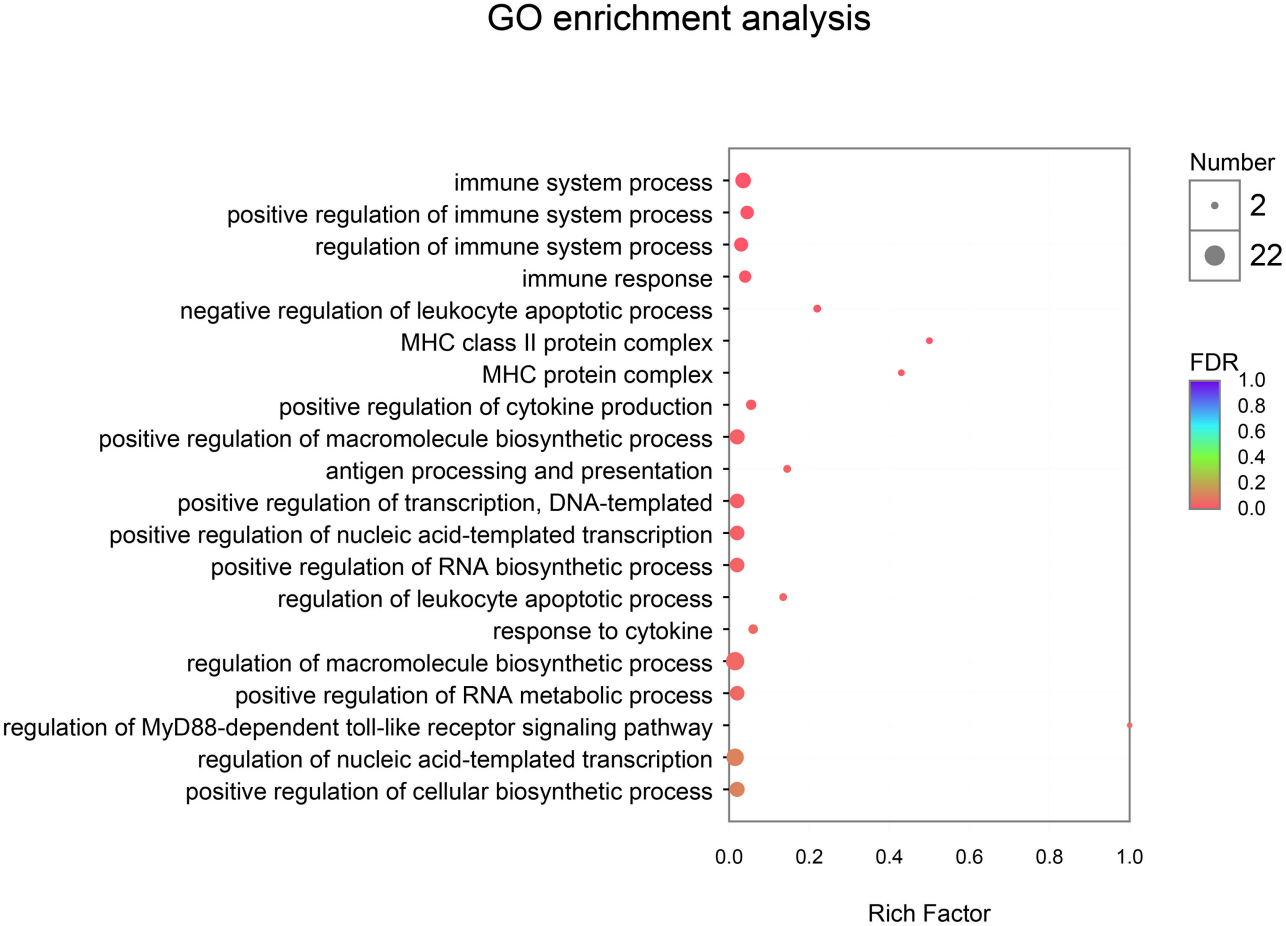
Gene ontology (GO) enrichment analysis between CON and EP_7000 treatment group of broilers. CON, dietary supplementation of *Enteromorpha* polysaccharides (EP) at 0 mg/kg; EP_7000, dietary supplementation of *Enteromorpha* polysaccharides (EP) at 7,000 mg/kg.

As described in Table 6 and Figure 3, 16 GO terms (*p*-corrected < 0.05) were significantly enriched. In GO enrichment analysis, the DEGs were divided into three main function groups, including biological processes, molecular functions, and cellular components. The result showed that the 16 terms significantly enriched the biological processes of the DEGs expressed in breast muscle. These were: immune system process, positive regulation of immune system process, immune response, negative regulation of leukocyte apoptotic process, positive regulation of cytokine production, positive regulation of macromolecule biosynthetic process, antigen processing and presentation, positive regulation of transcription, DNA-templated, positive regulation of nucleic acid-templated transcription, positive regulation of RNA biosynthetic process, positive regulation of RNA metabolic process, and regulation of MyD88-dependent toll-like receptors (TLRs) signaling pathway.

**Table 6:** The GO enrichment analysis between CON and EP_7000 groups of broiler chickens^a^.

**Number**	**Pathway ID**	**Pathway description**	***P*-corrected**
1	GO:0002376	Immune system process	0.000
2	GO:0002684	Positive regulation of immune system process	0.000
3	GO:0002682	Regulation of immune system process	0.002
4	GO:0006955	Immune response	0.004
5	GO:2000107	Negative regulation of leukocyte apoptotic process	0.004
6	GO:0001819	Positive regulation of cytokine production	0.016
7	GO:0010557	Positive regulation of macromolecule biosynthetic process	0.026
8	GO:0019882	Antigen processing and presentation	0.026
9	GO:0045893	Positive regulation of transcription, DNA-templated	0.028
10	GO:1903508	Positive regulation of nucleic acid-templated transcription	0.028
11	GO:1902680	Positive regulation of RNA biosynthetic process	0.034
12	GO:2000106	Regulation of leukocyte apoptotic process	0.036
13	GO:0034097	Response to cytokine	0.048
14	GO:0010556	Regulation of macromolecule biosynthetic process	0.048
15	GO:0051254	Positive regulation of RNA metabolic process	0.048
16	GO:0034124	Regulation of MyD88-dependent toll-like receptor signaling pathway	0.048

a *GO, Gene Ontology; CON, dietary supplementation of Enteromorpha polysaccharides (EP) at 0 mg/kg; EP7000, dietary supplementation of Enteromorpha polysaccharides (EP) at 7,000 mg/kg*.

**Figure 4:**
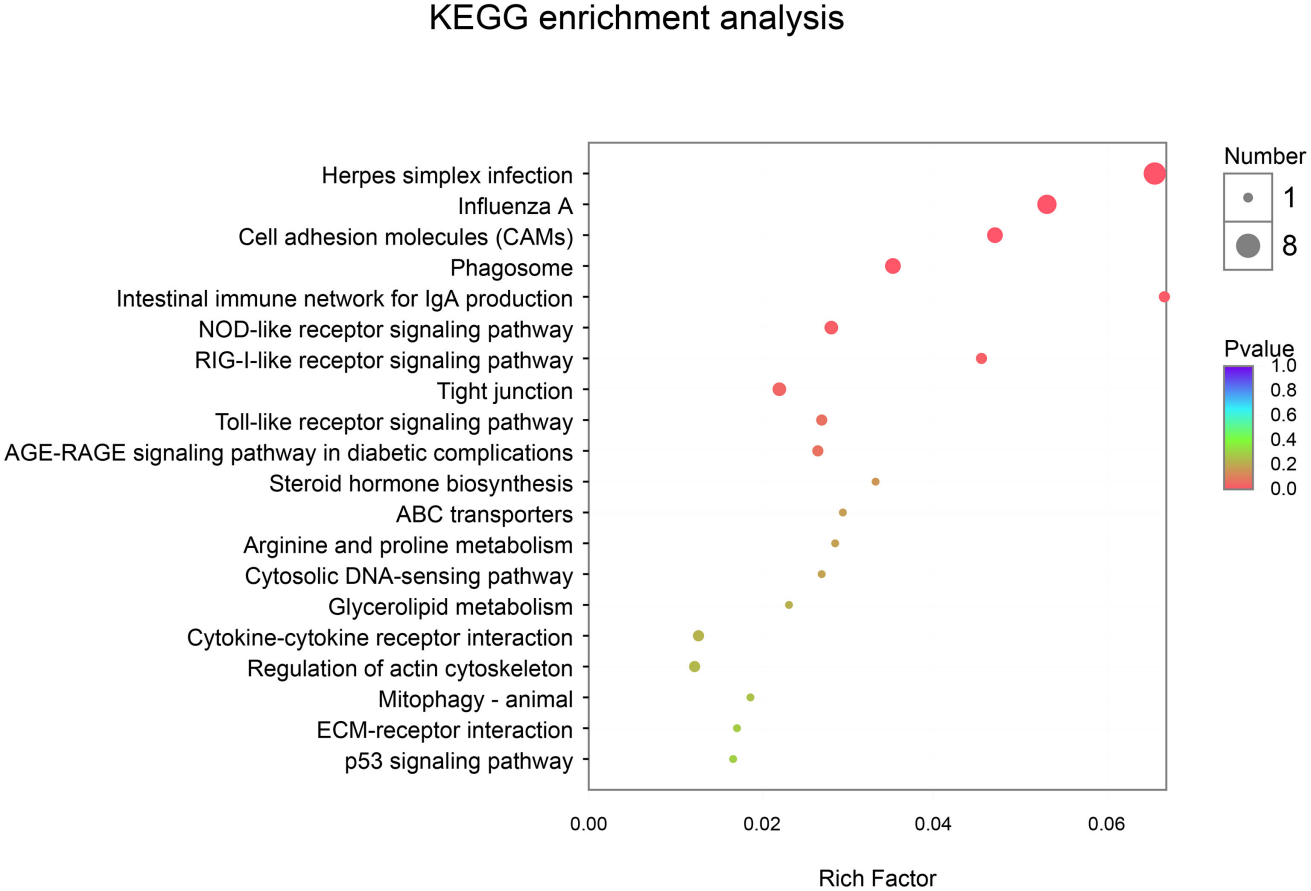
Kyoto Encyclopedia of Genes and Genomes (KEGG) enrichment analysis between CON and EP_7000 treatment group of broilers. CON, dietary supplementation of *Enteromorpha* polysaccharides (EP) at 0 mg/kg; EP_7000, dietary supplementation of *Enteromorpha* polysaccharides (EP) at 7000 mg/kg.

In addition, using KEGG enrichment analysis, the functions of transcripts and controls were mapped to pathways. As described in Table 7 and Figure 4, the KEGG pathway analysis (*p*-corrected < 0.05) showed that three pathways were significantly enriched and were associated with disease-related pathways, including Herpes simplex infection, Influenza A, and Cell adhesion molecules.

**Table 7:** The KEGG enrichment analysis between CON and EP_7000 groups of broiler chickens^a^.

**Number**	**Pathway ID**	**Pathway description**	***P*-corrected**
1	map05168	Herpes simplex infection	0.000
2	map05164	Influenza A	0.003
3	map04514	Cell adhesion molecules (CAMs)	0.054

a *KEGG, Kyoto Encyclopedia of Genes and Genomes; CON, dietary supplementation of Enteromorpha polysaccharides (EP) at 0 mg/kg; EP_7000, dietary supplementation of Enteromorpha polysaccharides (EP) at 7,000 mg/kg*.

## Discussion

Natural polysaccharides are widely used as functional feed additives in broiler nutrition, which can be used as immunomodulators to replace antibiotic growth promoters in food animals (28, 29). The present study's findings indicated that dietary supplementation of EP_7000 significantly improves breast muscle yield. Consistent with our results, Ou Yang et al. (24) observed that the inclusion of natural polysaccharides (1.0 and 1.5%) improved the breast muscle yield of broilers and suggested that it may be related to the ability of the polysaccharides to increase protein metabolism and decrease fat metabolism. Sun et al. (25) also found that the dietary supplementation of Astragalus polysaccharides (500 mg/kg) improved the breast muscle yield in broilers. Several possible mechanisms could explain the beneficial effects of these natural polysaccharides on breast muscle yield in broilers, including enhancement of the protein metabolism and resistibility. In addition, it may be related to the fact that natural polysaccharides significantly increased the absorption of nutrients from the intestine of broilers, which helps in improving the growth of broilers, thereby promoting breast muscle growth (22).

On the other hand, it may be that the natural polysaccharides could enhance the immunity and disease resistance of broilers, thus improving the growth and meat yield (22). However, the results of this study are in disagreement with the findings of Li et al. (29). They observed no significant differences in the breast muscle yield of broilers after feeding chitosan with different molecular weights. Also, Chen et al. (30) reported that the dietary inclusion of low molecular weight chitosan (< 2,374 Da) had no significant effects on breast muscle yield in broilers. The reasons for these inconsistent findings may be due to the different structures of polysaccharides and the age and feeding environment of broilers. It may also be associated with the purity and quantity of polysaccharides used in the diet. Also, the results of this study showed that the dietary EP at the supplemental level of 1,000, 2,500, 4,000, and 5,000 mg/kg had no significant effect on breast muscle yield, indicating that the supplemental dose of EP at 7,000 mg/kg is more effective, and the effect of dietary EP on carcass traits is dose dependent. However, the specific reason needs to be further investigated.

The mRNA transcriptome analysis was conducted to explore the potential molecular mechanism of dietary EP to increase the breast muscle yield in the present study. The results of transcriptome analysis showed that a total of 154 DEGs were identified between the CON and EP_7000 groups. The GO enrichment analysis of DEGs found that DEGs were mainly enriched in the immune-related signaling pathways. These genes enable the immune system to calibrate when the body is invaded by potential threats. Some genes activate and accelerate the production of cytokines and the apoptosis of white blood cells. Furthermore, it can modulate the expression of the MyD88-dependent TLRs signaling pathway. Similarly, Wang et al. (31) demonstrated that the dietary Astragalus polysaccharides enhanced the intestinal mucosa immunity of broilers by regulating immune-related signaling pathways (TLRs). Lycium barbarum polysaccharides were also reported to affect the TLRs-mediated signaling pathway, thereby improving the immune function and alleviating liver injury induced by carbon tetrachloride in mice (32). Also, Liu et al. (33) suggested that the Astragalus polysaccharides could enhance the immune function of mice by modulating TLRs/NF-κB signaling pathway. Li et al. (34) reported that the Astragalus polysaccharides exerted anti-inflammatory effects in LPS-infected Caco2 cells, also related to the TLRs-mediated immune signaling pathway. Similarly, Chen et al. (35) found that the natural polysaccharides (extracted from mulberry leaf) elevated the immunity by upregulating TLR7 levels in broilers.

Additionally, other genes are significantly enriched in biological processes related to cell and nucleic acid transcription regulation and macromolecular biosynthesis. These genes can activate or increase the frequency of RNA biosynthetic and nucleic acid-templated transcription (36). Moreover, it can improve the chemical reactions and pathways of macromolecules and the frequency of the RNA biosynthesis process. It is possible to promote RNA biosynthesis, accelerate the process of RNA transcription and metabolism and then through the positive regulation of macromolecular biosynthesis of breast muscle (37), thus promoting the development of breast muscle and increase the breast muscle yield in broilers. Meanwhile, the KEGG enrichment analysis results showed that the DEGs are enriched in signaling pathways related to viral infectious diseases and cell adhesion molecules. Cell adhesion molecules play an essential role in cell growth, development and differentiation, maintenance of normal tissue structure, inflammatory response, and immune response (38, 39). These immunity and cell adhesion-related signaling pathways may activate innate immunity and promote muscle cell development, thereby improving the muscle growth of broilers. In addition to the immunity and cell adhesion-related signaling pathways, it is worth noting that the Lipin1, Sestrin1 (SESN1) and fibroblast growth factor receptor 2 (FGFR2) genes of the DEGs are associated with protein synthesis and lipid metabolic process, which also contribute to the muscle growth. As known, the Lipin1 gene has an important regulatory role in lipolysis and reducing fat accumulation (40). The regulation of Lipin1 gene expression in lipid metabolism is accompanied by alteration of the protein synthesis process (41). Recently, Li et al. (41) confirmed that higher Lipin1 gene expression is beneficial to promote muscle development of broiler chickens. The SESN1 gene encodes Sestrin protein required for normal lifespan and its function in muscle cells to prevent muscle degeneration over a lifetime (42). A previous study reported that the SESN1 gene is involved in muscle development in chickens (43). Furthermore, the FGFR2-mediated cell proliferation and differentiation processes also play a crucial role in the regulation of muscle development, and the function of FGFRs in myogenesis and reduction of lipid deposition has been investigated both *in vitro* and *in vivo* (44). It has been suggested that FGF is a strong inhibitor of cell differentiation (45). The present study's findings showed that the inclusion of dietary EP improved the breast muscle yield which was related to the down-regulation of FGFR2 gene expression. Similar to our observations, Zheng et al. (45) found that the expression of FGFR2 negatively correlated with the muscle growth and development in broilers. Cui et al. (46) demonstrated that FGF7 and FGFR1 genes had down-regulation effects on breast muscle weight of broiler chickens. Therefore, in this study, dietary EP supplementation upregulated the expression of Lipin1 and SESN1 genes. In contrast, down-regulated expression of the FGFR2 gene might result in higher protein synthesis and lower lipid deposition, thus promoting muscle development. However, further verification studies are necessary to confirm these propositions.

## Conclusions

The results of this study suggest that the dietary supplementation of *Enteromorpha* polysaccharides at 7,000 mg/kg could promote the breast muscle yield of broilers. Further, the RNA-Seq analysis results showed that the differentially expressed genes were mainly enriched in immune-related signaling pathways, macromolecule biosynthetic, DNA-templated, RNA biosynthetic, protein synthesis, and lipid metabolic processes of broilers. Thus, the findings of the present study provided new insights for the dietary application of *Enteromorpha* polysaccharides as growth promoters and immune stimulants in broiler chickens.

## Data Availability Statement

The datasets presented in this study can be found in online repositories. The names of the repository/repositories and accession number(s) can be found here: https://www.ncbi.nlm.nih.gov/sra/PRJNA701498, PRJNA701498.

## Ethics Statement

The animal study was reviewed and approved by Animal Care Committee, Guangdong Ocean University.

## Author Contributions

W-CL, RJ, and BB: conceptualization, writing-review and editing. YZ and YG: analysis. YZ, S-JQ, and YG: data curation. YZ: writing-original draft preparation. W-CL and RJ: supervision. W-CL: project administration, methodology, and funding acquisition. All authors contributed to the article and approved the submitted version.

## Conflict of Interest

The authors declare that the research was conducted in the absence of any commercial or financial relationships that could be construed as a potential conflict of interest.

## References

[1] McAllister TA Wang Y Diarra MS Alexander T Stanford K. Challenges of a one-health approach to the development of alternatives to antibiotics. Anim Front. (2018) 8:10–20. 10.1093/af/vfy002 32002214 PMC6952028

[2] Jha R Das R Oak S Mishra P. Probiotics (Direct-fed microbials) in poultry nutrition and their effects on nutrient utilization, growth and laying performance, and gut health: a systematic review. Animals. (2020) 10:1863. 10.3390/ani10101863 33066185 PMC7602066

[3] Wang J Yang Z Celi P Yan L Ding X Bai S. Alteration of the anti-oxidant capacity and gut microbiota under high levels of molybdenum and green tea polyphenols in laying hens. Anti-Oxidants. (2019) 8:503. 10.3390/antiox8100503 31652580 PMC6826559

[4] Farag MR Elhady WM Ahmed SYA Taha HSA Alagawany M. *Astragalus* polysaccharides alleviate tilmicosin-induced toxicity in rats by inhibiting oxidative damage and modulating the expressions of HSP70, NF-kB and Nrf2/HO-1 pathway. Res Vet Sci. (2019) 124:137–48. 10.1016/j.rvsc.2019.03.010 30901666

[5] Yadav S Jha R. Strategies to modulate the intestinal microbiota and their effects on nutrient utilization, performance, and health of poultry. J Anim Sci Biotech. (2019) 10:2. 10.1186/s40104-018-0310-9 30651986 PMC6332572

[6] Wu S. Effect of dietary *Astragalus* membranaceus polysaccharide on the growth performance and immunity of juvenile broilers. Poult Sci. (2018) 97:3489–93. 10.3382/ps/pey220 29897509

[7] Long LN Kang BJ Jiang Q Chen JS. Effects of dietary *Lycium barbarum* polysaccharides on growth performance, digestive enzyme activities, anti-oxidant status, and immunity of broiler chickens. Poult Sci. (2020) 99:744–51. 10.1016/j.psj.2019.10.043 32029159 PMC7587896

[8] Zhang J Cai K Mishra R Jha R. In ovo supplementation of chitooligosaccharide and chlorella polysaccharide affect cecal microbial community, metabolic pathways, and fermentation metabolites in broiler chickens. Poult Sci. (2020) 99:4776–85. 10.1016/j.psj.2020.06.061 32988512 PMC7598314

[9] Guo Y Zhao ZH Pan ZY An LL Balasubramanian B Liu WC. New insights into the role of dietary marine-derived polysaccharides on productive performance, egg quality, anti-oxidant capacity, and jejunal morphology in late-phase laying hens. Poult Sci. (2020) 99:2100–7. 10.1016/j.psj.2019.12.032 32241495 PMC7587743

[10] Huang RL Yin YL Wu GY Zhang YG Li TJ Li LL. Effect of dietary oligochitosan supplementation on ileal digestibility of nutrients and performance in broilers. Poult Sci. (2005) 84:1383–8. 10.1093/ps/84.9.1383 16206559

[11] Li XJ Piao XS Kim SW Liu P Wang L Shen YB. Effects of chito-oligosaccharide supplementation on performance, nutrient digestibility, and serum composition in broiler chickens. Poult Sci. (2007) 86:1107–14. 10.1093/ps/86.6.1107 17495080

[12] Jiao Y Jha R Zhang WL Kim IH. Effects of chitooligosaccharide supplementation on egg production, egg quality and blood profiles in laying hens. Indian J Anim Res. (2019) 53:1199–204. 10.18805/ijar.B-881

[13] Li J Cheng Y Chen Y Qu H Zhao Y Wen C. Dietary chitooligosaccharide inclusion as an alternative to antibiotics improves intestinal morphology, barrier function, anti-oxidant capacity, and immunity of broilers at early age. Animals. (2019) 9:493. 10.3390/ani9080493 PMC6719223 31357589

[14] Hoeck VV Sonawane M Sanchez ALG Dosselaer IV Buyens C Morisset D. Chromium propionate improves performance and carcass traits in broilers. Anim Nutr. (2020) 6:480–7. 10.1016/j.aninu.2020.03.005 33364464 PMC7750789

[15] Liu WC Yuan YL Sun CY Balasubramanian B Zhao ZH An LL. Effects of dietary betaine on growth performance, digestive function, carcass traits, and meat quality in indigenous yellow-feathered broilers under long-term heat stress. Animals. (2019) 9:506. 10.3390/ani9080506 31370305 PMC6720770

[16] Bai H Bao Q Zhang Y Song Q Liu B Zhong L. Effects of the rearing method and stocking density on carcass traits and proximate composition of meat in small-sized meat ducks. Poult Sci. (2020) 99:2011–6. 10.1016/j.psj.2019.09.006 32241485 PMC7587699

[17] Sehoon P Hee YS Wook JC Chul MK Ki KD Wook JK. RNA-seq profiling of microdissected glomeruli identifies potential biomarkers for human iga nephropathy. American J Physiol Renal Physiol. (2020) 319:F809–21. 10.1152/ajprenal.00037.2020 32954852

[18] Liu S Lin L Jiang P Wang D Xing Y. A comparison of RNA-Seq and high-density exon array for detecting differential gene expression between closely related species. Nucleic Acids Res. (2011) 39:578–88. 10.1093/nar/gkq817 20864445 PMC3025565

[19] Sudhagar A Kumar G El-Matbouli M. Transcriptome analysis based on RNA-Seq in understanding pathogenic mechanisms of diseases and the immune system of fish: a comprehensive review. Int J Mol Sci. (2018) 19:245. 10.3390/ijms19010245 29342931 PMC5796193

[20] Jin HL Xu NJ Yan XJ. Research progress on the bioactive compounds of seaweed *Enteromorpha*. Marine Sci. (2011) 35:100–6. 10.1097/RLU.0b013e3181f49ac7

[21] Li QQ Wang CM Luo J Lv XZ Wang L Luo FB. Effects of dietary *Enteromorpha prolifera* polysaccharide on growth performance and immune function of broilers. China Poult. (2017) 39:24–8. 10.16372/j.issn.1004-6364

[22] Liu WC Guo Y Zhao ZH Jha R Balasubramanian B. Algae-derived polysaccharides promote growth performance by improving anti-oxidant capacity and intestinal barrier function in broiler chickens. Front Vet Sci. (2020) 7:1–10. 10.3389/fvets.2020.601336 33344535 PMC7738339

[23] Guo Y Balasubramanian B Zhao ZH Liu WC. Marine algal polysaccharides alleviate aflatoxin B1-induced bursa of Fabricius injury by regulating redox and apoptotic signaling pathway in broilers. Poult Sci. (2021) 100:844–57. 10.1016/j.psj.2020.10.050 33518138 PMC7858151

[24] Ou Yang KH Xiong XW Wang WJ Hu Y Lai YK Wu DK. Effects of water-soluble alfalfa polysaccharide on growth performance, carcass quality, growth hormone and insulin-like growth factor-1 gene expressions of broilers. Chinese J Anim Nutr. (2014) 26:1272–8. 10.3969/j.issn.1006-267x

[25] Sun B Xiao HY Zhou MC Luo Y He ZF Pan GY. Effect of Astragalus polysacharide in diets on slaughter performance and meat quality of broilers. Acta Ecologiae Animalis Domastici. (2015) 36:26–9. 10.3969/j.issn.1673-1182

[26] National Research Council. Nutrient Requirements of Poultry. 9th ed. Natl Acad Press, Washington, DC (1994).

[27] Teng PY Kim WK. Roles of prebiotics in intestinal ecosystem of broilers. Front Vet Sci. (2018) 5:245. 10.3389/fvets.2018.00245 30425993 PMC6218609

[28] Echeverry H Alizadeh M Yitbarek A Slominski B Rodriguez-Lecompte JC. Yeast cell wall polysaccharides enhanced expression of T helper type 1 and 2 cytokines profile in chicken B lymphocytes exposed to LPS challenge and enzyme treatment. Brit Poult Sci. (2021) 62: 125–30. 10.1080/00071668.2020.1817328 32875814

[29] Li QP Wang RL Pan W Zhao YZ Jiang GL Huang XL. Effects of different molecular weight chitosans on growth, slaughter performance, meat quality and nutrient metabolism of frizzled chickens. J Henan Agri Sci. (2015) 44:128–51. 10.15933/j.cnki.1004-3268

[30] Chen JW He XP Wang RL Liang CP Wang ZY Liu GQ. Effects of different molecular weight chitosan oligosaccharides on growth, lipid content, anti-oxidant activity and slaughter performance of Chinese yellow-feathered chickens at the age of 0 to 4 weeks. Chinese Poult Sci. (2020) 3:9–14.

[31] Wang X Li Y Shen J Wang S Yao J Yang X. Effect of Astragalus polysaccharide and its sulfated derivative on growth performance and immune condition of lipopolysaccharide treated broilers. Int J Biol Macromol. (2015) 76:188–94. 10.1016/j.ijbiomac.2015.02.040 25748840

[32] Chen X Yuan L Du J Zhang C Sun H. The polysaccharide from the roots of Actinidia eriantha activates raw264.7 macrophages via regulating microrna expression. Int J Biol Macromol. (2019) 132:203–12. 10.1016/j.ijbiomac.2019.03.158 30914371

[33] Liu T Zhang M Niu H Liu J Ma R Wang Y. Astragalus polysaccharide from Astragalus Melittin ameliorates inflammation via suppressing the activation of TLR-4/NF-κB p65 signal pathway and protects mice from CVB3-induced virus myocarditis. Int J Biol Macromol. (2019) 126:179–86. 10.1016/j.ijbiomac.2018.12.207 30586589

[34] Li YL Xu YJ Pan C Ren ZZ Yang XJ. TRIF is essential for the anti-inflammatory effects of Astragalus polysaccharides on LPS-infected Caco2 cells. Int J Biol Macromol. (2020) 159:832–8. 10.1016/j.ijbiomac.2020.05.005 32387604

[35] Chen X Yang H Jia J Chen Y Wang J Chen H. Mulberry leaf polysaccharide supplementation contributes to enhancing the respiratory mucosal barrier immune response in Newcastle disease virus–vaccinated chicks. Poult Sci. (2021) 100:592–602. 10.1016/j.psj.2020.11.039 33518112 PMC7858170

[36] Tompkins YH Su S Velleman SG Kim WK. Effects of 20(S)-hydroxycholesterol on satellite cell proliferation and differentiation of broilers. Poult Sci. (2020) 100:474–81. 10.1016/j.psj.2020.10.032 33518099 PMC7858162

[37] Hynes RO. Cell adhesion: old and new questions. Trends Genet. (1999) 15:M33–7. 10.1016/S0168-9525(99)01891-0 10611678

[38] Harjunpää H Llort Asens M Guenther C Fagerholm SC. Cell adhesion molecules and their roles and regulation in the immune and tumor microenvironment. Front Immunol. (2019) 10:1078. 10.3389/fimmu.2019.01078 31231358 PMC6558418

[39] Kim J Lee YJ Kim JM Lee SY Bae MA Ahn JH. PPARγ agonists induce adipocyte di?erentiation by modulating the expression of Lipin-1, which acts as a PPARγ phosphatase. Int J Biochem Cell Biol. (2016) 81:57–66. 10.1016/j.biocel.2016.10.018 27780754

[40] Ma X Wang L Shi Z Chen W Yang X Hu Y. Mechanism of continuous high temperature affecting growth performance, meat quality, and muscle biochemical properties of finishing pigs. Gene Nutri. (2019) 14:23. 10.1186/s12263-019-0643-9 31367261 PMC6657146

[41] Li D Pan Z Zhang K Yu M Yu D Lu Y. Identification of the di?erentially expressed genes of muscle growth and intramuscular fat metabolism in the development stage of yellow broilers. Genes. (2020) 11:244. 10.3390/genes11030244 32110997 PMC7140879

[42] Li Z Xu Y Lin Y. Transcriptome analyses reveal genes of alternative splicing associated with muscle development in chickens. Gene. (2018) 676:146–55. 10.1016/j.gene.2018.07.027 30010040

[43] Mitchell DL DiMario JX. Bimodal, reciprocal regulation of fibroblast growth factor receptor 1 promoter activity by BTEB1/KLF9 during myogenesis. Mol Biol Cell. (2010) 21:2780–7. 10.1091/mbc.E10-04-0290 20554758 PMC2912362

[44] Velleman SG Song Y. Development and growth of the avian pectoralis major (breast) muscle: function of syndecan-4 and glypican-1 in adult myoblast proliferation and differentiation. Front Physiol. (2017) 8:577. 10.3389/fphys.2017.00577 28848451 PMC5550705

[45] Zheng Q Zhang Y Chen Y Yang N Wang XJ Zhu D. Systematic identification of genes involved in divergent skeletal muscle growth rates of broiler and layer chickens. BMC Genom. (2009) 10:87. 10.1186/1471-2164-10-87 19232135 PMC2656524

[46] Cui HX Liu RR Zhao GP Zheng MQ Chen JL Wen J. Identification of differentially expressed genes and pathways for intramuscular fat deposition in pectoralis major tissues of fast-and slow-growing chickens. BMC Genom. (2012) 13:213. 10.1186/1471-2164-13-213 22646994 PMC3420248

